# Building the future health workforce: outreach pathways and the Holistic Ecosystem Model

**DOI:** 10.3389/fmed.2026.1863786

**Published:** 2026-07-08

**Authors:** Leila Amiri, Jonathan J. Wisco, Bei Zhang, Pamela C. Gibson, Nana Sartania

**Affiliations:** 1 Larner College of Medicine, University of Vermont, Burlington, VT, United States; 2 Boston University Chobanian and Avedisian School of Medicine, Boston, MA, United States; 3 School of Medicine Dentistry and Nursing, University of Glasgow, Glasgow, United Kingdom

**Keywords:** ecosystem approach to health professions access, health equity, health professions education, medical workforce diversity, outreach, widening access and participation

## Abstract

Healthcare workforce shortages and inequities in access to medical education remain persistent global challenges. Expanding participation in health professions education has become a central strategy for strengthening healthcare systems and improving population health outcomes. This perspective applies the Holistic Ecosystem Model (HES) as its central analytical framework to examine outreach initiatives across the United States, the United Kingdom, Australia, and Singapore. The HES conceptualizes pathways into medicine as dynamic systems shaped by four interacting layers: the learner pathway, educational structures, institutional systems, and community and policy environments. Viewing outreach through this ecosystem lens reveals why piecemeal interventions so often fall short–and what more coordinated, systemic strategies might achieve.

## 1 Introduction

The healthcare workforce shortage is a major obstacle to delivering effective health services and places a significant burden on healthcare education. Insufficient numbers of trained professionals limit access to care, increase provider workload, and exacerbate health disparities (1–3). Beyond overall workforce numbers, the composition and geographic distribution of physicians remain equally critical: in many countries, the physician workforce does not reflect the diversity of the populations it serves (4, 5), and rural and underserved communities face disproportionate shortages. Socioeconomic, geographic, and cultural barriers further widen the gaps in access to effective care.

These disparities are the downstream products of structural inequities that shape educational opportunities long before students consider medical school. Unequal school resources, limited mentorship, financial barriers, and disparities in academic preparation compound across time, determining who ultimately reaches the starting line (6). To address these substantial gaps, significant efforts have been made to diversify the workforce by expanding educational opportunities, with outreach programs playing a vital role (7, 8). Yet these programs are too often designed in isolation, disconnected from the institutional systems and policy environments that ultimately govern whether students succeed.

This paper applies the Holistic Ecosystem Model (HES) as its central analytical lens (9). The HES conceptualizes pathways into medicine not as linear sequences of individual achievement, but as dynamic ecosystems shaped by four interacting layers: the learner pathway, educational structures, institutional systems, and community and policy environments. The framework is introduced before the evidence because it changes how the evidence should be read. The effectiveness of any outreach initiative cannot be understood apart from the ecosystem surrounding it.

## 2 The Holistic Ecosystem Model

**FIGURE 1:**
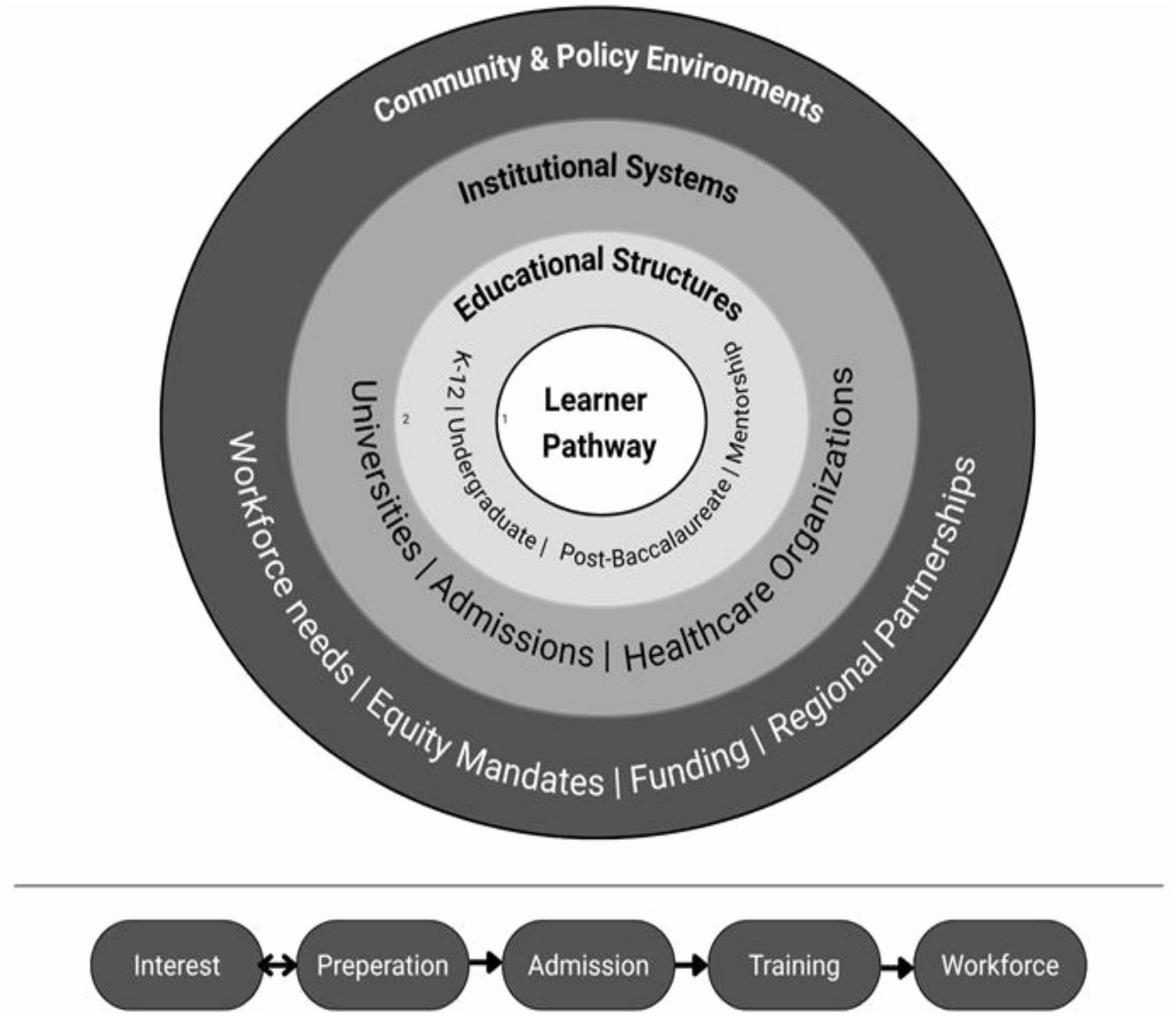
The Holistic Ecosystem Model (HES): four interacting layers shaping pathways into medicine.

The HES provides the conceptual architecture for this paper’s analysis (9). Rather than viewing pathways into medicine as linear sequences of individual achievement, the HES frames them as dynamic ecosystems shaped by the interaction of multiple layers of structural influence. The HES organizes these influences into four concentric layers, each shaping the layers within it (Figure 1).

Layer 1 – Learner pathway: the individual’s progression and development from early interest through preparation, admission, training, and professional practice. This trajectory is not fixed; it is continuously shaped, supported, or undermined by the surrounding layers.

Layer 2 – Educational structures: K-12 outreach, undergraduate preparation, post-baccalaureate programs, and mentorship networks. Most outreach energy is directed here, but effectiveness depends on how well these structures connect to the layers beyond them.

Layer 3 – Institutional systems: universities, admissions processes, and healthcare organizations. These systems govern access, determining which preparations are recognized and who receives support once enrolled. Outreach that prepares students to adapt their practices will struggle to produce durable change.

Layer 4 – Community and policy environments: workforce planning priorities, equity mandates, funding structures, and community partnerships. This outermost layer sets the conditions in which all inner layers operate.

A well-functioning pathway ecosystem is one in which these layers interact productively: outreach programs align with institutional admissions practices, institutional priorities respond to community workforce needs, and policy environments provide stable funding. The central question for each program and national context examined below is not what it does, but how it connects with the surrounding ecosystem–and where the gaps lie.

## 3 Why outreach matters: barriers across the ecosystem?

Understanding why outreach matters requires tracing the barriers learners face at each layer of the HES across the full developmental arc. Outreach is a process by which institutions connect with a community for any combination of the following purposes: philanthropy, recruitment, mandate, resource sharing, education, community building, public service, righting inequities, and partnership. In the context of academic institution-community outreach, a university may be interested in sharing resources and providing educational opportunities to improve public health outcomes, while the community may be interested in accessing resources it cannot generate on its own.

At the learner pathway layer, disparities in academic confidence, career awareness, and professional identity emerge early and are self-reinforcing. Students from underrepresented backgrounds often lack the informal knowledge networks, family connections to medicine, peer groups navigating similar aspirations, and teachers who can facilitate exposure to clinical environments that students from more privileged backgrounds take for granted. Ample research demonstrates that a demographically diverse healthcare provider workforce is associated with improved patient outcomes (10–12) and that physicians from underrepresented backgrounds are more likely to practice in underserved communities. Yet a perpetual problem is that healthcare facilities struggle to supply a provider demographic that matches their community.

At the educational structures layer, unequal school resources compound initial disadvantages. Limited access to Advanced Placement coursework, the absence of health professions career advising, and underfunded science education widen preparation gaps before students encounter formal pathway programs. In the early 2000s, significant effort was placed on increasing K-12 children’s interest in science, but by the 2010s, the needle on workforce diversity had not moved meaningfully. Recent evidence, however, points to the importance of opportunity cost, the cost of starting, staying on, and completing the healthcare career pathway, as the primary obstacle. Outreach partnerships should therefore focus on minimizing the opportunity cost of pursuing healthcare careers.

At the institutional layer, admissions processes frequently fail to recognize the preparation and resilience of applicants from non-traditional backgrounds. Without deliberate institutional adaptation, even students who successfully navigate the educational layer may encounter a wall at the point of admission (8). At the community and policy layer, workforce planning tends to respond to shortages reactively rather than shaping the upstream pipeline proactively, and project-dependent funding creates chronic instability in programs that require sustained engagement to produce measurable change.

## 4 Categories of outreach programs: interventions across the ecosystem

Outreach initiatives operate at different layers of the HES and take many forms across educational stages. Four broad categories reflect distinct points of intervention in the ecosystem.

K-12 initiatives (Educational Structures Layer) plant the earliest seeds of interest and professional identity. Programs can begin as early as kindergarten, though middle and high school students are the most common audience. In the United States, initiatives like the AAMC Pathway Programs, Meyerhoff Scholars Program, and Anatomy Academy (13, 14) offer mentorship, clinical exposure, and college preparation. In the United Kingdom, WAMSS sessions and NHS-funded Medical Schools Council Summer Schools target hard-to-reach pupils. In Australia, the University of Western Australia’s Rural High School Medical Program supports rural students as they navigate UCAT preparation and university entry.

Pre-medical college programs (Educational Structures and Institutional Layers) prepare students for institutional entry and begin shifting expectations about who belongs in medicine. US programs, including Summer Health Professions Education Program (SHPEP, formerly SMDEP) and the Perry Initiative, provide career guidance, clinical exposure, and mentorship to historically underrepresented students (15, 16). In the United Kingdom, the Sutton Trust’s Pathways to Medicine program, along with organizations such as MelaninMedics and The Aspiring Medics Support, offer personalized admissions guidance and peer mentoring.

Post-baccalaureate pathways (Educational Structures and Institutional Layers) have been shown to increase diversity among matriculating medical students by supporting applicants from historically underrepresented backgrounds who need additional academic preparation (17). US programs in Michigan, Southern Illinois, and New Jersey offer intensive coursework and MCAT preparation. In the United Kingdom, Gateway and similar programs serve the equivalent function, with evidence showing comparable outcomes for Gateway and standard entry students (18).

Community-based and regional pipelines (Community and Policy Layer) represent outreach designed around workforce destination rather than learner demographics alone. US Rural Health Scholars initiatives combine training, mentorship, and financial support to encourage practice in underserved areas (6, 19). In Scotland, Reach and SWAP support disadvantaged students applying to high-tariff professions. In Australia, the RHMT program works with 22 universities and 28 regional training hubs to increase rural practice exposure (20), while a government-mandated 25% rural student intake quota connects institutional admissions directly to community workforce needs (21).

## 5 Best practices and innovations: building connections across the ecosystem

The literature notes that successful outreach elements include early intervention, mentorship, community engagement, centering student voices and lived experiences, maintaining longevity and sustainability, training facilitators, and measuring outcomes. Viewed through the HES lens, these features are not merely best practices; they are mechanisms by which programs create connections across ecosystem layers.

Early and sustained engagement builds a scaffold across the educational structures layer at every transition point. Longitudinal programs that accompany students from elementary school through medical school application are paramount, given that disparities in educational opportunities often begin early. Programs such as Anatomy Academy (13, 14) and the Community Health Scholars summer program (22) demonstrate how structured curricula can engage younger learners while connecting them to community health contexts.

Layered mentorship networks linking high school, undergraduate, and medical students operate at the intersection of the learner and educational layers, ensuring role models are accessible at every educational transition. Co-designing educational opportunities based on the needs of the community, including school systems, teachers, parents, and learners, is foundational to effective mentorship.

Academic enrichment and professional preparation address the preparation gap at the educational structures layer while building institutional-layer readiness. MCAT (Medical College Admissions Test) and UCAT (Universal Clinical Aptitude Test) preparation, research experiences, clinical shadowing, and structured advising ensure students are not only exposed to healthcare careers but also equipped to compete for them (17).

Community partnership and sustainability require garnering both philosophical and financial support from stakeholders who understand the value of providing an outreach pathway. When institutions demonstrate long-term commitment, the relationship with community partners evolves from intervention to investment–a process captured by social accountability frameworks developed specifically for health professional education (23). Diversifying the workforce remains a primary goal, given compelling data showing that a diverse workforce provides better healthcare to those with whom they share identities.

Measuring outcomes should examine not only participants’ immediate reflections but also long-term outcomes, tracking whether there was an impact on the number of individuals entering health-related careers. Australia has been tracking data for years and has developed a network to share data across programs. Literature is rich with innovative ways to engage students, including leveraging digital technologies to reach rural students or maintain engagement during periods when in-person outreach is challenging (24).

Support beyond admission is essential; outreach that ends at admission leaves the learner pathway incomplete. Academic support networks, peer mentoring, and affinity groups within medical schools are as much a part of the outreach ecosystem as pre-admission programs. The ecosystem must extend into medical training. Admission without retention is not equity.

## 6 Comparative national perspectives: ecosystem configurations

Different national contexts represent distinct configurations of HES layers. Table 1 summarizes these configurations; the analysis below identifies the alignment strengths and gaps each reveals.

**TABLE 1:** Outreach ecosystems supporting health workforce pathways across countries.

Country/ region	Primary policy driver	Typical outreach structures	Workforce goal	Ecosystem characteristics
United States	Workforce diversity and equitable access	Pipeline programs, summer academies, mentorship networks, post-baccalaureate programs, AHEC partnerships	Increase representation of underrepresented populations; address regional shortages	Multi-stakeholders: universities, federal agencies, nonprofits, community partners
United Kingdom	Widening participation in higher education	Mentoring initiatives, contextual admissions, national summer schools, charity-led advising	Increase participation from lower socioeconomic backgrounds	Strong national alignment: universities, charities, government education policy
Australia	Rural and regional workforce development	Rural pipeline programs, rural clinical schools, scholarship programs, and regional training hubs	Increase the physician workforce in rural and remote communities	Government-supported regional infrastructure linking universities and health systems
Singapore	Centralized workforce planning within a competitive academic system	Mentorship programs, early exposure initiatives, and academic enrichment	Maintain high academic standards while broadening access	Highly coordinated national education and workforce planning

The United States operates a decentralized multi-stakeholder ecosystem–broad in reach but structurally weak in coordination. Because programs are frequently designed and funded independently, connections between educational structures, institutional admissions, and community workforce needs remain implicit rather than deliberately built (6). The United Kingdom illustrates what stronger cross-layer integration achieves: contextual admissions practices represent an institutional-layer adaptation explicitly linked to educational-layer programs (25), and the UKWPMED partnership–in which seven medical schools recognize each other’s outreach interventions and offer contextual admissions accordingly–shows how institutional coordination amplifies educational-layer impact. Australia anchors its ecosystem geographically around workforce destination rather than learner demographics, with the RHMT program and the 25% rural student intake quota connecting institutional admissions directly to community-layer needs (20, 21). Singapore’s centrally coordinated system produces the tightest institutional-to-policy alignment of any context examined here, though high coordination may reduce responsiveness to heterogeneous learner needs (26).

Across these contexts, the HES reveals a consistent pattern: ecosystem effectiveness depends not on any single layer but on the quality of connections between them.

## 7 Cross-cutting challenges: ecosystem gaps

Despite the growth of outreach initiatives globally, several structural challenges persist. The HES framework clarifies their nature, each of which can be understood as a gap or misalignment between ecosystem layers.

Funding instability represents a failure at the community-policy layer. Outreach is expensive, and financial resources are scarce. In the United States, federal mechanisms like the NIH R25 provide some support but tend to fund supplies rather than protected faculty time. The ASPBP-NBME partnership offers the highest available direct costs for outreach activities, but salaries remain excluded. Dependence on project-cycle funding creates structural fragility; programs requiring years of sustained engagement cannot achieve scale under short-term award cycles.

Staff bandwidth compounds this problem at the institutional level. Faculty engagement in outreach is frequently not recognized within academic promotion structures, creating a structural disincentive that undermines the educational-layer programs faculty make possible. It is asking a great deal of outreach directors to devote time to publishing their work when funding mechanisms do not provide protected time to do so.

Evaluation and longitudinal tracking gaps persist because following participants across multiple educational stages demands a cross-institutional data infrastructure that most systems have not built. Without longitudinal data, it is difficult to know which ecosystem interventions produce durable change and where gaps between layers are largest. Describing the process of measuring and analyzing outcome measures is key to securing funding and to building the evidence base for advocacy at the federal level.

Preparation without institutional connection leaves students in the gap between educational and institutional layers. Admissions reform and outreach design must be developed in coordination–a program that successfully prepares students but deposits them at an admissions process that does not recognize their preparation has not completed the ecosystem intervention.

Admission without retention similarly represents an incomplete intervention. The learner pathway does not end at enrollment, and the outreach ecosystem must extend into medical training through academic support networks, peer mentoring, and affinity groups that help students successfully complete their training.

## 8 Conclusions and future directions

Outreach initiatives represent a critical component of global strategies to address healthcare workforce shortages and inequities in medical education. Programs across diverse national contexts demonstrate that early exposure, mentorship, academic preparation, and institutional collaboration can meaningfully expand participation in healthcare careers. But the HES framework makes clear that the central challenge of workforce development is not program design; it is ecosystem development.

The question is not whether any individual program works. Many do. The question is whether the programs, institutions, policies, and community structures surrounding individual learners are configured to support sustained progress across the full arc of the learner pathway–from early interest through professional practice in communities that need them. This framing also reorients persistent tensions in the field: the supply-versus-demand debate is recast as a question of ecosystem coordination; the perceived conflict between equity and excellence is resolved when institutions build strong connections across all four layers, producing both. The risk of overcompensation in widening participation efforts is best managed through community co-design and longitudinal outcome tracking rather than by narrowing ambition.

Future efforts should be oriented around four priorities, each corresponding to a dimension of ecosystem strengthening. First, coordinated ecosystem development: the most significant gains will come not from expanding isolated programs but from deliberately building the connections between them. Cross-sector coordination should be treated as a design requirement, not an aspiration. Second, a longitudinal data infrastructure that tracks participants from early exposure through workforce entry will identify where ecosystem gaps are largest. Australia’s data-sharing network offers a model worth examining for adaptation in other national contexts. Third, technology and geographic reach: virtual mentoring platforms, digital learning environments, and mobile educational initiatives can extend the ecosystem’s reach into geographically isolated communities, addressing community-layer workforce needs where physical infrastructure is difficult to sustain (24). Fourth, international collaboration: no single system has fully solved the challenge of coordinating across all HES layers, making cross-national partnerships for sharing best practices and underutilized resources.

The HES framework offers a way of thinking about outreach not as a collection of programs to be implemented but as an ecosystem to be cultivated. Combining outreach with career pathways and inclusive admission enhances impact, increases workforce diversity, and ensures sustainable efforts to meet growing healthcare demands. Building the future health workforce requires attending to every layer of that ecosystem–and to the quality of the connections between them. Strengthening these interconnections represents a long-term investment in healthcare workforce sustainability, educational opportunity, and global health equity.

## Data Availability

The original contributions presented in this study are included in this article/supplementary material, further inquiries can be directed to the corresponding authors.
